# Attention-based Transformer-LSTM architecture for early diagnosis and staging of early-stage Parkinson’s disease using fNIRS data

**DOI:** 10.3389/fnagi.2025.1677722

**Published:** 2025-10-10

**Authors:** Huan Wang, Hujun Wang, Shuyan Qie, Congxiao Wang, Nan Li, Hanming Wang

**Affiliations:** Department of Rehabilitation, Beijing Rehabilitation Hospital, Capital Medical University, Beijing, China

**Keywords:** Parkinson’s disease, functional near-infrared spectroscopy, deep learning, Transformer, LSTM

## Abstract

**Background:**

Parkinson’s disease (PD) is a progressive neurodegenerative disorder requiring early diagnosis and accurate staging for optimal treatment outcomes. Traditional clinical assessments have limitations in objectivity and reproducibility.

**Objective:**

To develop and validate an Attention-based Transformer-LSTM hybrid deep learning model (ATLAS-PD) for classifying early-stage PD patients (H&Y stages 1–2) and healthy controls using functional near-infrared spectroscopy (fNIRS) data.

**Methods:**

This cross-sectional study enrolled 240 participants: 80 healthy controls, 80 H&Y stage 1 PD patients, and 80 H&Y stage 2 PD patients. fNIRS data were collected during a pegboard task using a 22-channel system covering prefrontal cortex regions. To address task-specific bias, a pilot complementary gait imagery task was performed on a subset of 60 participants (20 per group), with additional ROC AUC analysis. The ATLAS-PD model was compared with traditional machine learning algorithms including Support Vector Machine, Random Forest, K-Nearest Neighbors, and Back-Propagation Neural Network. McNemar’s test and bootstrap resampling were conducted to assess superiority. Interpretability analysis was conducted using permutation importance to quantify channel contributions, with regional aggregation and channel ranking to identify neurophysiologically relevant patterns. Additionally, t-SNE (t-distributed Stochastic Neighbor Embedding) dimensionality reduction was applied to visualize the feature space clustering.

**Results:**

The ATLAS-PD model achieved an accuracy of 88.9% (95% CI: 0.808–0.970), demonstrating superior robustness and generalization compared to traditional approaches. While SVM showed higher accuracy (92.6, 95% CI: 0.869–0.983) on the test set, it exhibited significant performance degradation under noise conditions (accuracy dropped to 45.2% at *σ* = 0.3). ATLAS-PD maintained 80.09% accuracy at the same noise level, indicating superior clinical applicability. The model achieved AUC values of 0.99, 0.78, and 0.88 for healthy controls, H&Y stage 1, and H&Y stage 2 groups, respectively. For the gait imagery task, macro-average AUC was 0.723, confirming model robustness across tasks. Statistical tests confirmed ATLAS-PD significantly outperformed baselines (*p* < 0.05). Interpretability analysis using permutation importance and attention weight visualization revealed the model primarily utilizes bilateral frontal polar cortex signals, with channels CH01, CH04, CH05, and CH08 showing highest importance scores. t-SNE visualizations further demonstrated distinct clustering of healthy controls from PD groups, with partial overlap between H&Y stages 1 and 2, reflecting the disease continuum.

**Conclusion:**

ATLAS-PD provides an objective, non-invasive tool for early PD diagnosis and staging in H&Y stages 1–2. The inclusion of complementary tasks and statistical validations enhances its clinical applicability. Future studies should validate the model’s performance in more advanced PD stages to enhance clinical applicability.

## 1 Introduction

Parkinson’s disease (PD) is a common neurodegenerative disorder characterized by motor symptoms including tremor, muscle rigidity, and bradykinesia, affecting over 10 million patients globally (Yang et al., 2020; Dorsey et al., 2018). With an aging population, the incidence of PD continues to rise, making early diagnosis and disease staging crucial for optimizing treatment strategies and delaying disease progression (Zhang et al., 2005; Marras et al., 2018). Traditional diagnosis relies on clinical assessments such as the Unified Parkinson’s Disease Rating Scale (UPDRS) and the Hoehn–Yahr (H&Y) staging system (Hoehn and Yahr, 1967; Fahn, 1987). However, these standards have inherent limitations: first, symptom manifestation exhibits latency, with diagnosis typically occurring when the disease has progressed to relatively advanced stages (Tolosa et al., 2021; Beach et al., 2009); second, early PD symptoms overlap with other neurological disorders, leading to high misdiagnosis rates (Rizzo et al., 2016; Adler et al., 2014); third, while scales like UPDRS provide standardized assessment frameworks, evaluator subjectivity and patient state fluctuations affect result reproducibility (Goetz et al., 2008; Martínez-Martín et al., 1994). Recent advances in PD biomarker research have identified several promising candidates including alpha-synuclein aggregates in cerebrospinal fluid, dopamine transporter imaging, and olfactory dysfunction assessments (Parnetti et al., 2019; Kalia and Lang, 2015). However, these approaches often require invasive procedures, expensive equipment, or lack sufficient sensitivity for early-stage detection (Berg et al., 2015).

Recent advances in neuroimaging technology have played increasingly important roles in PD research (Weiller et al., 2006). Functional near-infrared spectroscopy (fNIRS), as a non-invasive, portable neuroimaging technique, reflects neural activity by measuring cortical hemodynamic changes and has demonstrated tremendous potential in PD research (Ferrari and Quaresima, 2012; Pinti et al., 2020). fNIRS offers advantages including high temporal resolution, relative insensitivity to motion artifacts, and the ability to conduct measurements in natural environments, making it an ideal tool for investigating the neural mechanisms underlying motor and cognitive dysfunction in PD patients (Scholkmann et al., 2014; Bonilauri et al., 2022). While various neuroimaging modalities have been explored for PD diagnosis, each presents distinct advantages and limitations. Functional magnetic resonance imaging (fMRI) offers excellent spatial resolution but requires patients to remain motionless in confined spaces, limiting its applicability for motor task assessments (Poldrack et al., 2017). Electroencephalography (EEG) provides superior temporal resolution but suffers from poor spatial localization and high susceptibility to motion artifacts during motor tasks (Michel and Murray, 2012). Positron emission tomography (PET) and single-photon emission computed tomography (SPECT) can visualize dopaminergic function but involve radiation exposure and high costs (Marek et al., 2011). In contrast, fNIRS combines reasonable spatial resolution for cortical regions, excellent temporal resolution, and high tolerance to motion artifacts, making it particularly suitable for naturalistic motor assessments in clinical populations (Ferrari and Quaresima, 2012). Multiple studies have utilized fNIRS to explore cortical activation patterns and functional connectivity abnormalities in PD patients during motor or cognitive tasks. For instance, research has identified delayed hemodynamic changes in the primary motor cortex during fine motor tasks and reduced resting-state functional connectivity in PD patients (Nieuwhof et al., 2016). Other studies have observed altered prefrontal cortex activation during dual-task processing using fNIRS (Guevara et al., 2024a; Vitorio et al., 2017). Existing research demonstrates that the prefrontal cortex (PFC) in PD patients exhibits abnormal activation patterns during motor control and cognitive functions. For example, early-stage PD patients may display PFC hyperactivation as a compensatory mechanism, while mid-to-late stages show hypoactivation (Galna et al., 2015). However, most fNIRS studies are limited to descriptive analyses, lacking fine classification of multiple staging categories (Clark et al., 2014).

With rapid developments in artificial intelligence technology, machine learning and deep learning methods have been widely applied to medical image analysis and disease diagnosis (Miotto et al., 2018). In the PD field, machine learning classification models based on fNIRS data have become research hotspots, aiming to achieve early PD diagnosis and severity assessment through brain activity pattern analysis (Hui et al., 2024). Traditional machine learning algorithms such as support vector machines (SVM), random forest (RF), and k-nearest neighbors (KNN) have been attempted for fNIRS data classification with some progress (Nilashi et al., 2017; Castillo-Barnes et al., 2018). However, fNIRS data possesses characteristics of high dimensionality, time series nature, and nonlinearity, which traditional machine learning methods may struggle to fully capture in their complex spatiotemporal dynamics (Lamba et al., 2022). Deep learning, particularly Long Short-Term Memory (LSTM) networks and Transformer models, demonstrates significant advantages in processing sequential data and capturing long-range dependencies, providing new insights for fNIRS data analysis (Vaswani et al., 2017; Lipton et al., 2015). LSTM, through its gating mechanisms, can effectively learn long-term dependencies in time series data, while Transformer’s self-attention mechanism can process information from different positions in sequences in parallel, capturing global dependencies (Devlin et al., 2019; Hochreiter and Schmidhuber, 1997). Combining the advantages of these two models holds promise for further improving the performance and robustness of fNIRS-based PD classification models.

Furthermore, the clinical translation of deep learning models in healthcare requires not only high accuracy but also interpretability to gain clinician trust and regulatory approval (Holzinger et al., 2017). Recent advances in explainable AI, including attention visualization and permutation importance methods, offer promising approaches to unveil the decision-making process of complex models (Adadi and Berrada, 2018). Moreover, dimensionality reduction techniques such as t-SNE (t-distributed Stochastic Neighbor Embedding) can enhance interpretability by visualizing high-dimensional feature spaces, providing a complementary approach to understand the neural patterns captured by deep learning models. By applying these techniques to fNIRS-based PD classification, we aim to identify neurophysiologically meaningful patterns that align with established understanding of PD pathophysiology, thereby bridging the gap between computational performance and clinical interpretability.

Nevertheless, existing PD early diagnosis research has certain limitations: first, many studies have small sample sizes (*n* < 100), resulting in poor model generalization (Vyas et al., 2022); second, robustness evaluation under noise interference is neglected, limiting clinical applications (Senturk, 2020); third, comparative analyses are often limited to single models, where SVM’s advantages in high-dimensional data may be offset by overfitting risks, while RF’s ensemble learning, though robust, lacks interpretability (Jha et al., 2019). This study aims to utilize fNIRS technology combined with the ATLAS-PD algorithm (Attention-based Transformer-LSTM Architecture for Staging Parkinson’s Disease) to classify and identify PD patients at different H&Y stages, comparing with traditional machine learning models. Our hypothesis is that this hybrid model can improve multi-class classification accuracy and demonstrate superior robustness. We hope to provide more accurate and reliable tools for early PD diagnosis, disease progression assessment, and treatment effect monitoring, thereby improving the quality of life for PD patients.

## 2 Materials and methods

### 2.1 Study design and subject selection

This study employed a cross-sectional design, including three different subject groups: PD H&Y stage 1 patient group, PD H&Y stage 2 patient group, and healthy control group. Each group included 80 subjects, totaling 240 participants. Detailed demographic and clinical characteristics of the subjects are shown in Table 1. Sample size calculation was conducted using G*Power 3.1.4 software based on *a priori* power analysis. Based on previous fNIRS-PD studies reporting medium effect sizes (Cohen’s *d* ≈ 0.3–0.5) (Bonilauri et al., 2022; Nieuwhof et al., 2016; Wang et al., 2024), we assumed a medium effect size (Cohen’s *f* = 0.30) for one-way ANOVA comparing three groups. With a statistical power of 0.80, *α* = 0.05, and three-group design, the minimum required sample size was calculated as 111 participants total. We selected 80 participants per group (240 total) to provide additional margin and account for potential dropouts. For deep learning model training, our sample size (*n* = 240) exceeds the minimum requirements suggested in similar neuroimaging studies (*n* > 200), ensuring adequate data for model training and validation without overfitting (Li X. et al., 2023; Eken et al., 2024; Lei et al., 2024).

**Table 1:** Baseline characteristics of subjects.

Characteristic	Healthy controls	PD-HY1 group	PD-HY2 group
Sample size	80	80	80
Age (years)	61.2 ± 8.0	65.4 ± 7.2	69.9 ± 5.7
Gender (male/female, %)	40.0/60.0	55.0/45.0	58.75/41.25
Height (cm)	163.2 ± 4.2	161.2 ± 2.5	166.4 ± 5.2
Body weight (kg)	61.6 ± 3.2	63.8 ± 2.1	62.1 ± 3.3
Disease duration (years)	N/A	2.5 ± 1.1	2.7 ± 1.3
UPDRS score	N/A	11.0 ± 3.9	22.7 ± 4.1
L-dopa equivalent doses (LEDs)	N/A	371.5 ± 159.5	379.1 ± 142.3

N/A indicates not applicable. L-dopa equivalent doses (LEDs) calculated according to standard conversion factors. Medications included: levodopa/carbidopa (78% of PD patients), dopamine agonists (45%), MAO-B inhibitors (23%), and COMT inhibitors (12%). All patients were in “ON” medication state during testing.

#### 2.1.1 Parkinson’s disease patients

The PD group in this study included 160 patients (91 males, 69 females), all diagnosed by neurological specialists at Beijing Rehabilitation Hospital, Capital Medical University, according to the International Movement Disorder Society clinical diagnostic criteria for PD (Postuma et al., 2015). The study employed a modified H&Y staging system for disease severity assessment. Among these, 80 patients were in H&Y stage 1 (44 males, 36 females), and 80 patients were in H&Y stage 2 (47 males, 33 females). Inclusion criteria included: (1) newly diagnosed primary PD patients without other disease history; (2) H&Y staging 1–2 (early-stage disease allowing reliable task performance); (3) right-handedness; (4) ability to cooperate in completing experimental tasks without significant motor limitations. Exclusion criteria included: (1) secondary parkinsonism; (2) history of cerebrovascular disease, neurosurgical procedures, or brain tumors; (3) history of alcohol or substance dependence; (4) severe cognitive dysfunction; (5) other severe neurological or psychiatric disorders. Withdrawal criteria were: (1) occurrence of serious adverse events; (2) failure to complete testing according to the established protocol; (3) voluntary withdrawal from the study. This study was limited to H&Y stages 1–2 for several reasons: (1) Early-stage patients can reliably perform pegboard tasks without confounding effects of severe motor impairment; (2) Early diagnosis and staging have greater clinical impact for treatment optimization; (3) Advanced-stage patients (H&Y 3–5) often have significant tremor, rigidity, and bradykinesia that would preclude reliable task performance and introduce substantial motion artifacts in fNIRS recordings. All patients received standard anti-Parkinsonian medication during the study period.

#### 2.1.2 Healthy control group

The healthy control group consisted of 80 subjects from staff and outpatient health examination individuals at Beijing Rehabilitation Hospital, Capital Medical University, including 32 males and 48 females, with ages matched to the PD group. Exclusion criteria included: (1) intracranial tumors, trauma, or other significant neurological diseases; (2) severe medical conditions; (3) inability to complete fNIRS examination; (4) history of psychiatric disorders or current use of medications affecting cognitive function.

### 2.2 Ethical approval and informed consent

This study was approved by the Ethics Committee of Beijing Rehabilitation Hospital, Capital Medical University (Ethics approval number: 2022bkky-029). All participants provided written informed consent after receiving detailed explanation of the research purpose, methods, potential risks, and benefits.

### 2.3 Data acquisition equipment

**Figure 1:**
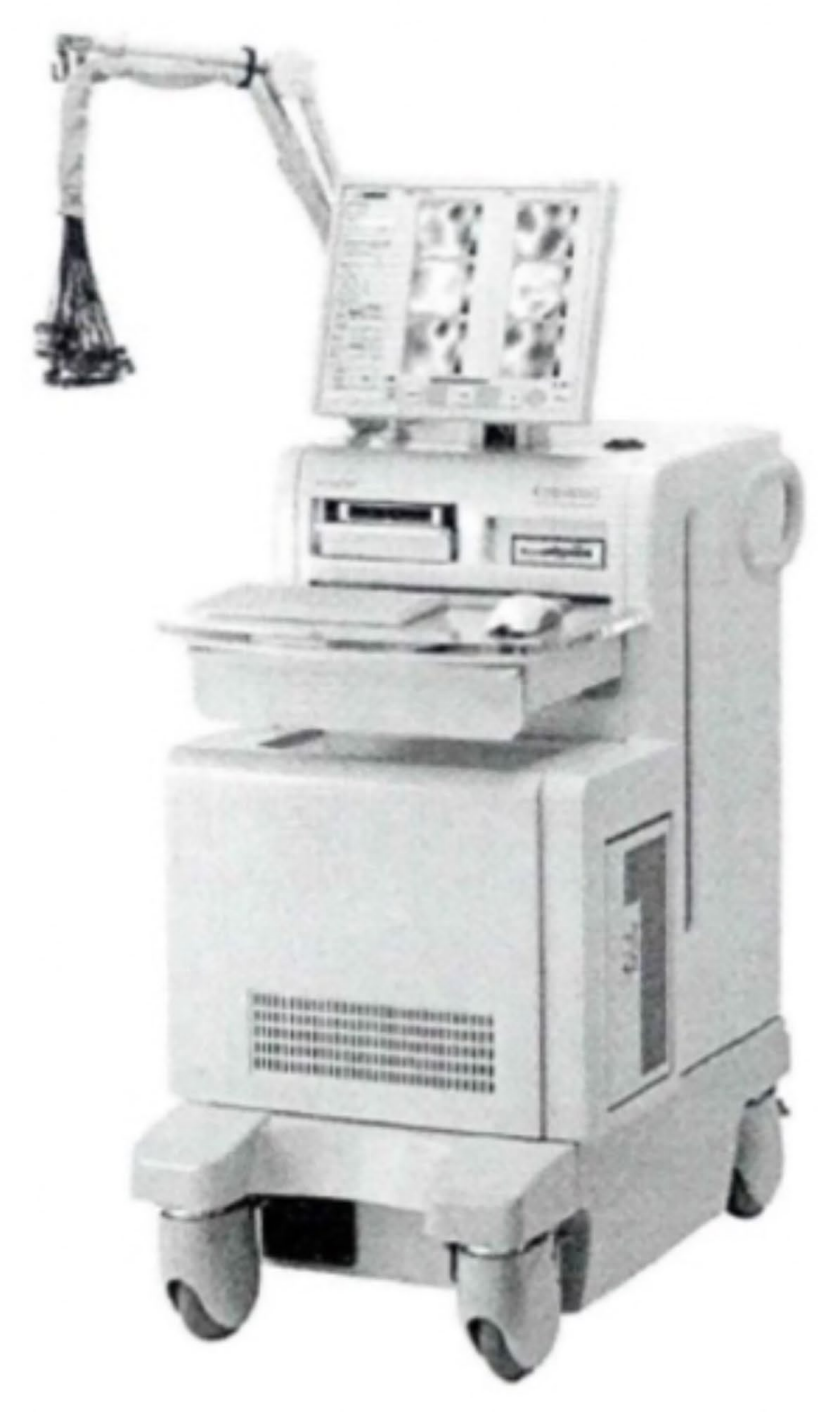
ETG-4000 device.

In this study, data were acquired using the ETG-4000 Optical Topography system, an fNIRS device, as shown in Figure 1. This device utilizes two wavelengths of near-infrared light (695 nm and 830 nm) transmitted to the scalp through emission optical fibers and received by detection optical fibers. The ETG-4000 can continuously measure hemoglobin concentration changes in multi-channel mode, calculating total hemoglobin concentration.

**Figure 2:**
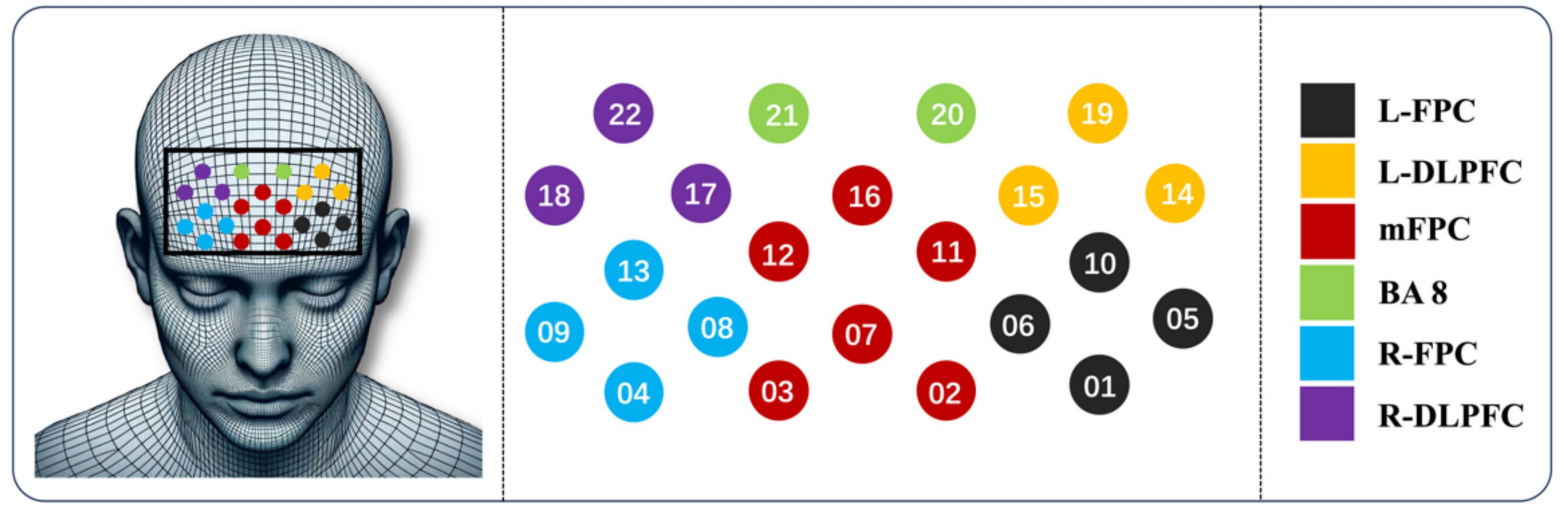
Optode and probe placement.

The experiment used a customized optode cap to measure participants’ prefrontal cortex regions. The probe holder was equipped with 8 emission optodes and 7 detection optodes (3 cm spacing), forming 15 probes and 22 channels (CH). Channel distribution strategically covered important cortical areas: CH01, CH05, CH06, CH10: left frontal polar cortex (L-FPC); CH04, CH08, CH09, CH13: right frontal polar cortex (R-FPC); CH02, CH03, CH07, CH11, CH12, CH16: medial frontal polar cortex (mFPC); CH14, CH15, CH19: left dorsolateral prefrontal cortex (L-DLPFC); CH17, CH18, CH22: right dorsolateral prefrontal cortex (R-DLPFC); CH20, CH21: Brodmann area 8 (BA8), as shown in Figure 2. These regions play crucial roles in cognitive function, decision-making, social cognition, complex problem-solving, and information integration across different brain areas (Ehlis et al., 2014). The sampling frequency was set to 100 Hz to ensure sufficient temporal resolution for capturing hemodynamic changes.

### 2.4 Experimental design and data collection

**Figure 3:**
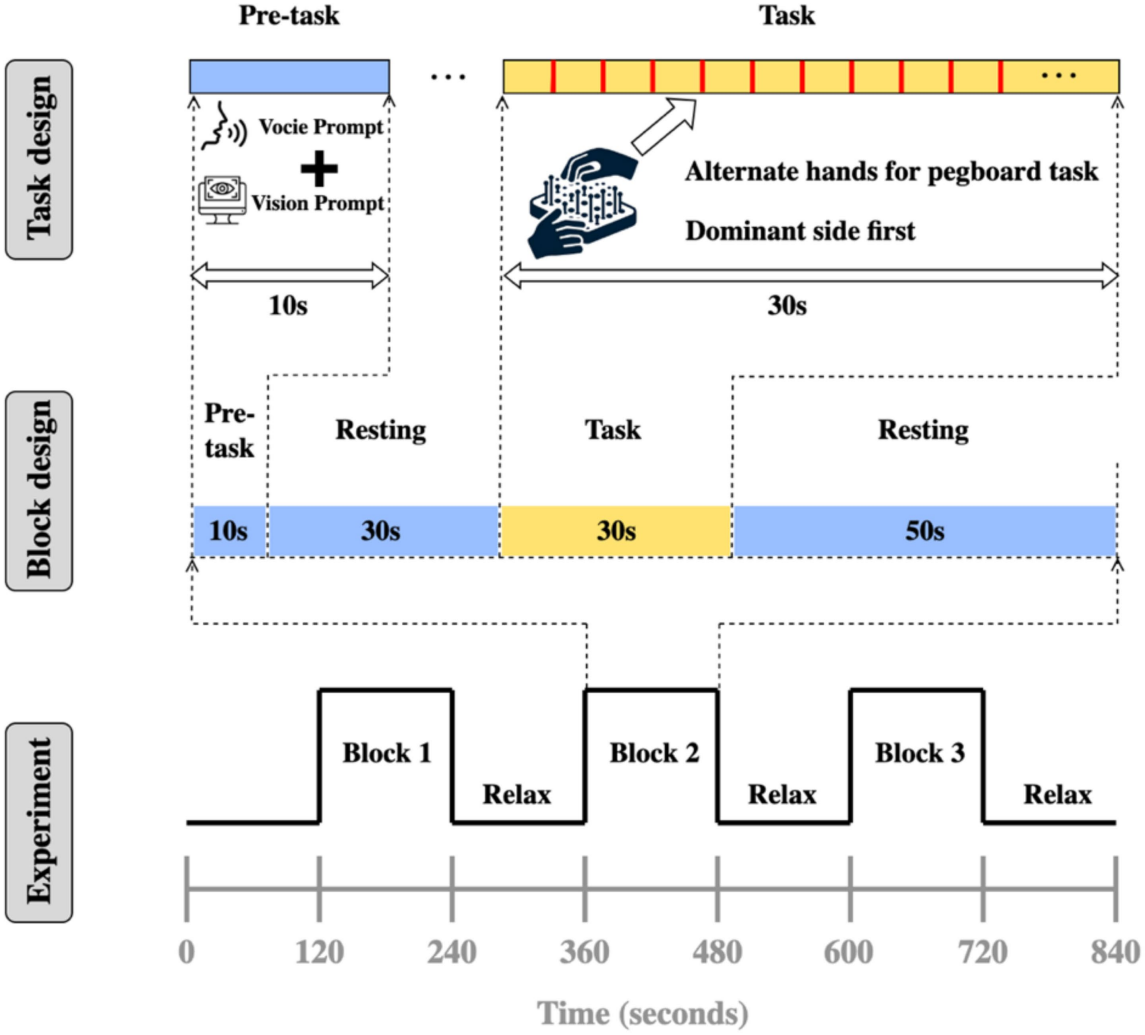
Experimental design.

The experiment employed a block design paradigm, with each test cycle including: pre-task phase (blank screen 10 s), rest phase (blank screen 30 s), task phase (task execution 30 s), rest phase (blank screen 50 s). The total experimental duration was 840 s (14 min), including multiple task-rest cycles, as illustrated in Figure 3. The 30-s task duration was selected based on established fNIRS hemodynamic response characteristics, where the main peak of hemodynamic response function (HRF) occurs around 5–6 s with a duration of approximately 8 s, followed by an undershoot peaking around 15 s (Machado et al., 2021). This duration aligns with successful fNIRS motor paradigms that have employed 20–30 s task blocks (Carius et al., 2016).

To mitigate task-specific bias associated with the pegboard task, a pilot complementary task involving gait imagery (mental simulation of walking) was conducted on a subset of 60 participants (20 healthy controls, 20 H&Y stage 1 PD patients, and 20 H&Y stage 2 PD patients). Participants were instructed to imagine walking at a comfortable pace while seated, with the same block design paradigm (30-s imagery phases). fNIRS data were collected using the same 22-channel setup.

**Figure 4:**
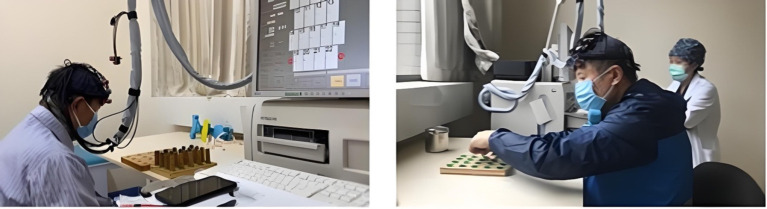
Participant testing procedure.

Data collection was conducted in a quiet, light-controlled environment. Participants were asked to relax for 5 min before the experiment to minimize hemodynamic responses induced by previous activities. During the experiment, all potential environmental disturbances were eliminated, participants were instructed to remain relaxed, avoid unnecessary movements or thinking, and sit comfortably in chairs. During the task phase, subjects were required to continuously complete pegboard tasks using both hands. This task required subjects to insert pegs into holes in the pegboard as quickly and accurately as possible, challenging their hand dexterity and coordination, as shown in Figure 4. According to recent research from institutions including the University of Florida and Northwestern University, pegboard tasks can provide objective, reliable data for tracking motor symptom progression in PD and atypical parkinsonism (Buard et al., 2022; Proud et al., 2020; Bezdicek et al., 2014). This is a practical, cost-effective measurement method that complements subjective clinical scales and expensive imaging techniques, providing a direct approach for efficacy assessment in clinical trials and research.

### 2.5 Data processing

Raw light intensity data were converted to optical density using the modified Beer–Lambert law. Motion artifacts were corrected using the Temporal Derivative Distribution Repair (TDDR) algorithm with standard deviation threshold of 3.0 and amplification factor of 0.5 (Fishburn et al., 2019). Complex artifacts were addressed using hybrid spline interpolation and wavelet filtering methods (Huang and Li, 2024). Channel quality was assessed using the Signal Quality Index (SQI) algorithm and Scalp Coupling Index (SCI) (Pollonini et al., 2014; Sappia et al., 2020). Channels with SQI < 2.5 or SCI < 0.5 were excluded. Signals were filtered using a 4th-order Butterworth low-pass filter with 0.1 Hz cutoff frequency following established guidelines (Pinti et al., 2020). Hemoglobin concentrations were calculated using the modified Beer–Lambert law with differential path length factor of 6.0 (Scholkmann et al., 2014). Baseline correction used 10-s pre-task mean subtraction, followed by Savitzky–Golay smoothing (polynomial order 3, window length 5) and channel-wise *Z*-score normalization. Optode positions were standardized using the 10–20 EEG system with anatomical landmarks. Channel locations were registered to MNI space using the NIRS-SPM toolbox (Ye et al., 2009). Data processing used MATLAB R2022b with NIRS_KIT package and MNE-Python functions.

### 2.6 Model construction

#### 2.6.1 Data preparation

The dataset matrix combined with subject categories formed a 240 × 22 × 84,000 three-dimensional tensor, where 240 represents the number of samples, 22 represents the number of channels, and 84,000 represents the number of time points. Subsequently, the dataset underwent standardization processing, including normalization, outlier handling, missing value management, and feature binarization. Data normalization employed the *Z*-score standardization method, ensuring data were on the same scale by subtracting the mean from each feature value and dividing by the standard deviation. Outliers were identified and handled using the Interquartile Range (IQR) method. For missing data, this study employed a multiple imputation approach to fill in missing values based on the values of other variables, maintaining data integrity and minimizing bias that missing data might introduce.

#### 2.6.2 Deep learning model selection

**Figure 5:**
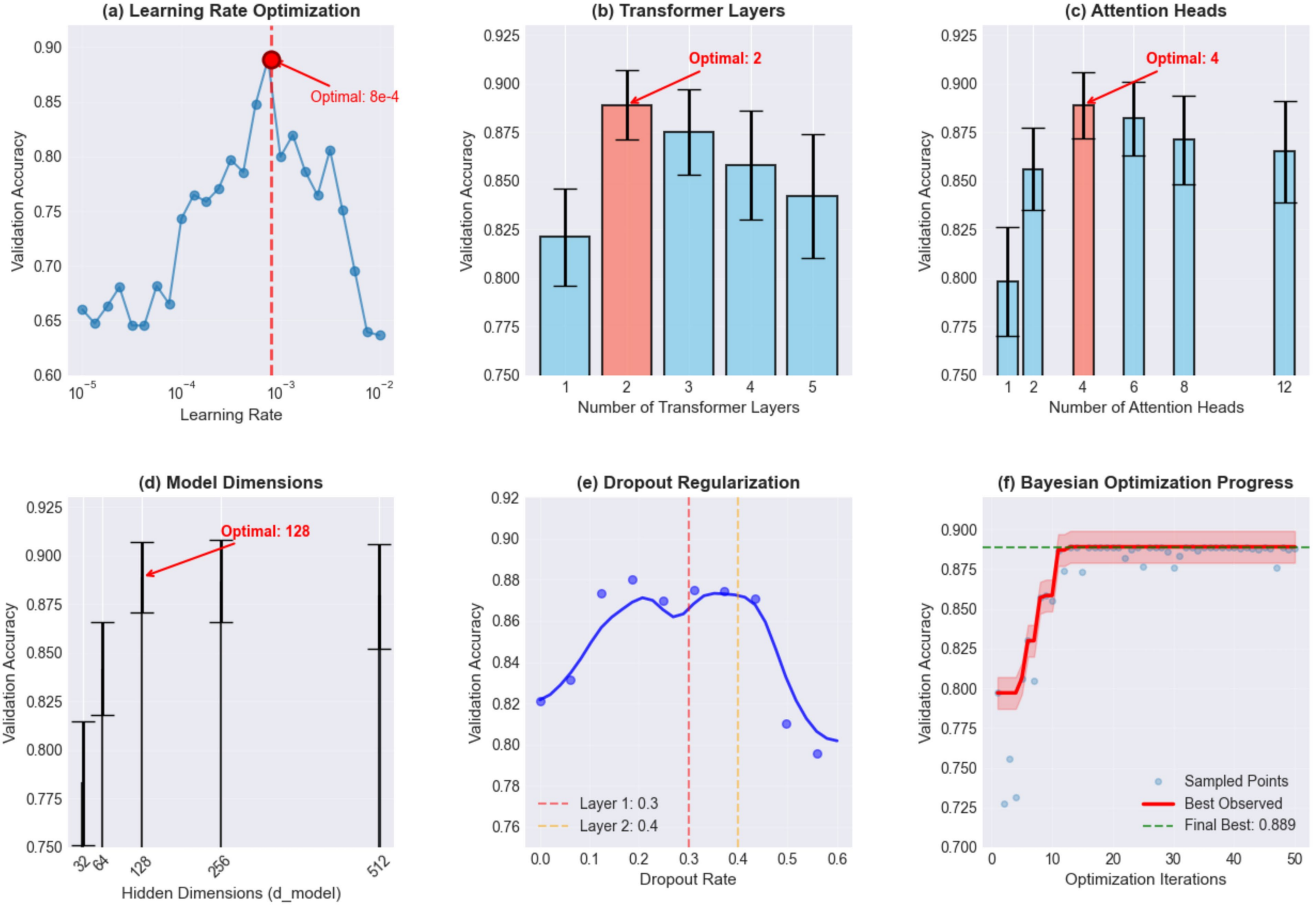
Hyperparameter optimization results **(a)** Learning rate optimization showing validation accuracy across different learning rates with optimal value at 8e-4. **(b)** Transformer layers optimization revealing optimal performance with 2 layers. **(c)** Attention heads analysis indicating optimal performance with 4 attention heads. **(d)** Model dimensions showing validation accuracy across different hidden dimensions with optimal value at 128. **(e)** Dropout regularization displaying the effect of different dropout rates on validation accuracy with optimal values of 0.3 and 0.4 for different layers. **(f)** Bayesian optimization progress tracking the convergence of the optimization process over 50 iterations, achieving a final best validation accuracy of 0.889.

This study selected LSTM networks as the primary deep learning model, based on the following considerations: First, fNIRS signals are essentially time series data containing temporal change information of brain hemodynamics. LSTM, through its unique gating mechanisms (input gate, forget gate, and output gate), can effectively capture long-term temporal dependencies, which is crucial for understanding cortical activation patterns in PD patients during motor task execution (Lipton et al., 2015). Second, LSTM can selectively retain or forget historical information, making it particularly suitable for processing long-range correlations and complex temporal dynamics in fNIRS signals (Tortora et al., 2020). To further enhance model performance, this study innovatively combined Transformer attention mechanisms with LSTM. This hybrid architecture design is based on the concept that Transformer’s multi-head self-attention mechanism can process information from different positions in sequences in parallel, capturing global dependencies, while LSTM focuses on modeling local temporal patterns (Vaswani et al., 2017; Wan et al., 2023). This complementarity enables the model to understand both overall patterns in fNIRS signals and capture subtle temporal changes, thereby more accurately identifying brain functional characteristics of PD patients with different severity levels. Hyperparameter optimization was conducted systematically using Bayesian optimization with Gaussian Process surrogate models over 50 iterations. The search space encompassed: Transformer layers [1–5], attention heads [1–12], learning rate [1 × 10^−5^ to 1 × 10^−2^] on log scale, hidden dimensions [64–512], and dropout rates [0.1–0.6]. Optimization used 5-fold cross-validation on the training set with Expected Improvement acquisition function. The optimal configuration achieved was: 2 Transformer layers, 4 attention heads, learning rate 8e-4, d_model = 128, dropout rates [0.3, 0.4] for different layers (Figure 5). These values are summarized in Table 2.

**Table 2:** Hyperparameters of each algorithm model.

Algorithm	Hyperparameters
LSTM	epochs: 120, lr: 0.002, hidden_size: [128, 64], dropout: [0.3, 0.4]
Transformer + LSTM	epochs: 120, lr: 0.0008, d_model: 128, nhead: 4, layers: 2
SVM	C: 0.8, gamma: scale, kernel: rbf
BP-NN	hidden_layers: (80, 40), alpha: 0.02, max_iter: 180
RF	n_estimators: 80, max_depth: 8, min_samples_split: 8
KNN	n_neighbors: 7, weights: distance, algorithm: kd_tree

#### 2.6.3 Traditional machine learning model comparison

**Figure 6:**
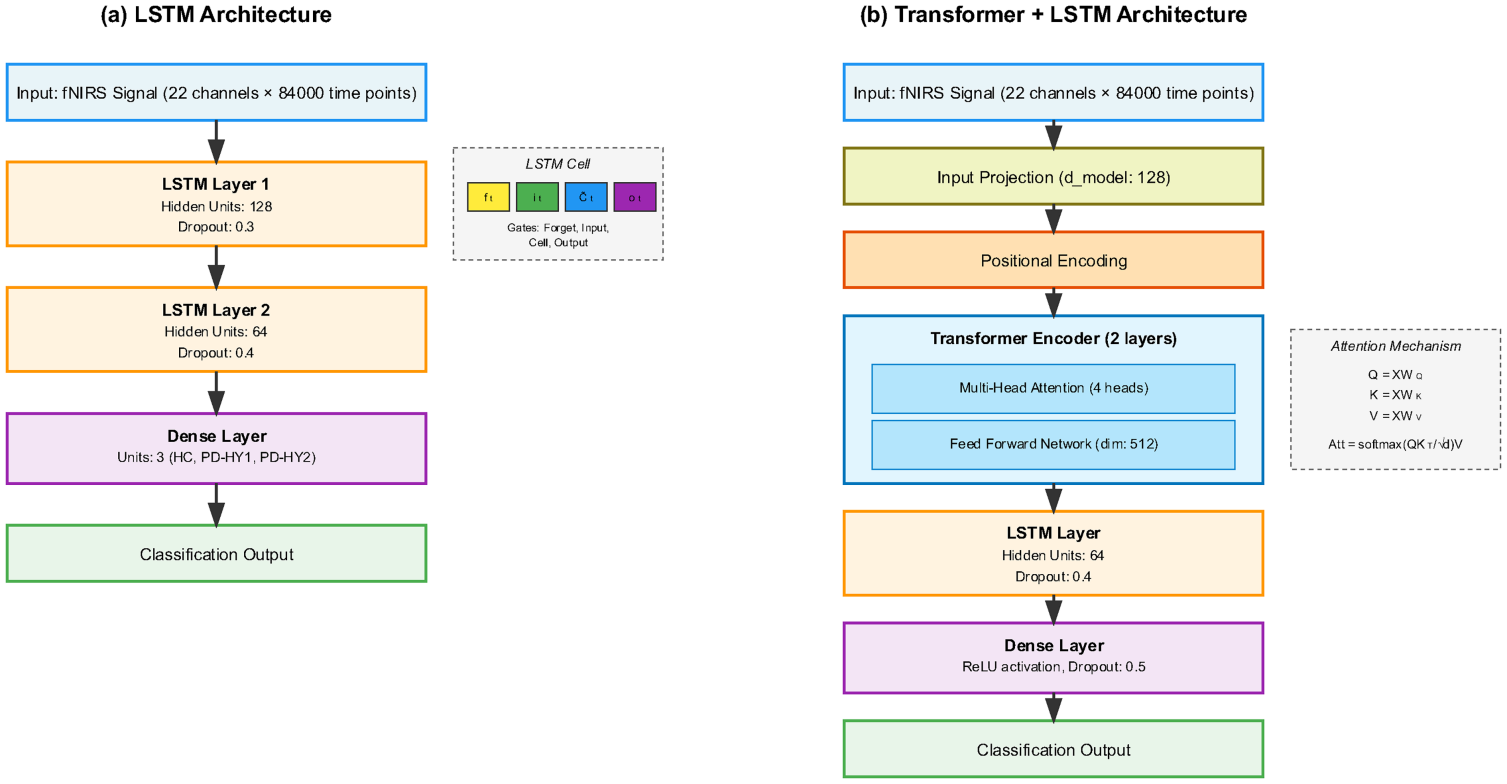
Neural network architecture comparison **(a)** LSTM architecture showing the baseline model with two LSTM layers (128 and 64 hidden units) followed by a dense classification layer. **(b)** Transformer + LSTM architecture displaying the hybrid model with input projection, positional encoding, transformer encoder with multi-head attention mechanism, LSTM layer, and final classification output.

To comprehensively evaluate deep learning model performance, this study selected four representative traditional machine learning algorithms for comparative analysis. SVM was chosen for its excellent performance in high-dimensional spaces and its ability to handle nonlinear problems, particularly suitable for processing multi-channel fNIRS data. BP Neural Network (MLP), as a classic feedforward neural network, can learn nonlinear mapping relationships in data, providing a benchmark comparison for deep learning methods. RF reduces overfitting risk through ensemble learning strategies, providing robust classification performance, while its feature importance assessment function helps identify key fNIRS channels. KNN algorithm, based on instance-based learning methods, is simple and intuitive, reflecting local structural characteristics of data. These four algorithms cover different machine learning paradigms, providing a comprehensive comparison benchmark for evaluating deep learning model advantages. Specific hyperparameter configurations for each model are detailed in Table 2 (see Figure 6).

#### 2.6.4 Model training strategy

For deep learning models, the dataset was split into training (70%, *n* = 168), validation (15%, *n* = 36), and testing (15%, *n* = 36) sets using stratified sampling to maintain class balance. Early stopping was implemented with patience of 20 epochs based on validation loss to prevent overfitting. Regularization techniques included dropout (rates: 0.3–0.4 for different layers), batch normalization, and L2 weight regularization (*λ* = 0.001). Model checkpointing saved the best-performing model based on validation accuracy. For traditional machine learning models, 5-fold stratified cross-validation was performed on the combined training and validation sets (*n* = 204), with the independent test set (*n* = 36) reserved for final evaluation. This approach ensures unbiased performance estimation and fair comparison between deep learning and traditional methods (Hastie et al., 2005).

### 2.7 Model evaluation metrics

For comprehensive evaluation of model performance, this study included calculation of confusion matrices and receiver operating characteristic (ROC) curves. The confusion matrix provides detailed information about true positives, false positives, true negatives, and false negatives, aiding in understanding the model’s performance in differentiating between categories. The ROC curve, its “Area Under the Curve” (AUC), and the *F*_1_-score provide quantitative measures of a model’s overall performance and are vital tools for assessing classifier efficacy (Fawcett, 2006). These metrics are extensively utilized as comprehensive evaluation indicators in various diagnostic models. The ROC curve plots the true positive rate against the false positive rate at various threshold settings, enabling visualization of a classifier’s performance across different thresholds. The AUC represents the degree to which the model can distinguish between classes; a higher AUC value indicates better model performance. The *F*_1_-score, a harmonic mean of precision and recall, is particularly useful in situations where an even balance between false positives and false negatives is critical. It is a single metric that combines the sensitivity and precision of the classifier, offering a balanced view of its performance, especially in cases of imbalanced datasets. These tools are integral in providing a holistic assessment of the classifier’s accuracy and reliability in diagnostic models.

### 2.8 Model robustness validation

To evaluate model stability in practical applications, this study designed noise injection experiments to test model robustness. Six different noise levels were set (*σ* = 0.0, 0.1, 0.2, 0.3, 0.4, 0.5), adding Gaussian white noise to test data and observing changes in model accuracy trends. The noise addition formula was:


$$X\_\text{noisy}=X\_\text{original}+N(0,\sigma^{2})$$

where *N*(0, *σ*^2^) represents a Gaussian distribution with mean 0 and variance *σ*^2^. Model anti-noise capability was quantified by calculating the area under the noise robustness curve (AUC_robustness).

### 2.9 Model interpretability analysis

To address the black-box nature of deep learning models and provide clinically interpretable insights, this study employed permutation importance methods to quantify the contribution of each fNIRS channel to model classification performance. Importance scores were normalized to the [0, 1] interval, with higher values indicating greater channel contribution to model decisions. The importance scores of 22 channels were aggregated according to their anatomical regions, calculated as the average importance score of constituent channels. Additionally, all channels for each model were ranked in descending order by importance scores to identify the key channels most relied upon by each model. Additionally, t-SNE was applied to reduce the dimensionality of features extracted from the ATLAS-PD model’s penultimate layer (van der Maaten and Hinton, 2008). The analysis utilized perplexity values of 30 and 50, to generate 2D and 3D projections, density contours, and comparative visualizations.

### 2.10 Statistical analysis

Statistical analysis was completed using Python 3.11 and SPSS 26.0. For data that are normally distributed and have homogeneous variances, independent sample *t*-tests were utilized for intergroup comparisons; for datasets not adhering to normal distribution, we applied non-parametric tests (Mann–Whitney *U* test) to ensure accurate statistical analysis. McNemar’s test was used to assess the paired differences in classification errors on the test set. Bootstrap resampling (with *n* = 1,000 iterations) was employed to estimate the 95% confidence intervals for accuracy differences and calculate the *p*-values for the superiority test. A significance level of *p* < 0.05 was set, indicating that differences are statistically significant. All analyses were performed on a workstation with Intel i9-10900K CPU, 32GB RAM, and NVIDIA RTX 3080 GPU. Software versions: Python 3.11.5, PyTorch 2.0.1, scikit-learn 1.3.0, MATLAB R2022b, SPSS 28.0. Random seeds were set (Python: 42, PyTorch: 123) for reproducibility.

## 3 Results

### 3.1 Post-hoc power analysis

Post-hoc power analysis was conducted based on the observed effect sizes in our study. For age differences between groups, ANOVA revealed a significant main effect [*F*(2, 237) = 30.63, *p* < 0.001, *η*^2^ = 0.205], representing a large effect size (Cohen’s *f* = 0.508). The achieved statistical power for detecting age differences was >0.99, indicating more than adequate sensitivity. For UPDRS scores comparing PD-HY1 and PD-HY2 groups, the observed effect size was Cohen’s *d* = 2.93 (very large effect), with achieved power >0.99. Similarly, disease duration differences showed adequate effect detection with power >0.85. These results confirm that our sample size (*n* = 240) provided sufficient statistical power to detect clinically meaningful differences between groups, validating the appropriateness of our sample size calculation.

### 3.2 Dataset distribution characteristics

**Figure 7:**
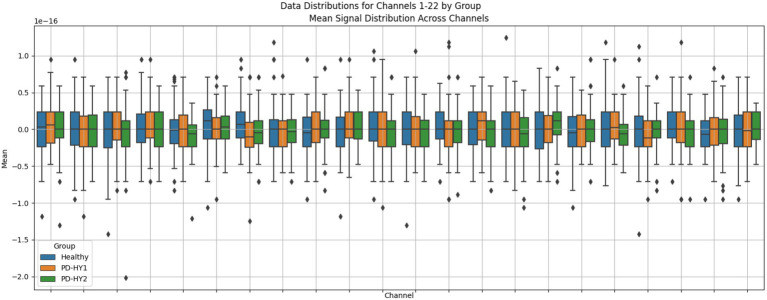
Data distribution.

The preprocessed dataset showed relatively balanced distribution characteristics across groups, as shown in Figure 7. Average signal intensities from channels CH01 to CH22 displayed intergroup differences, with data generally trending toward normal distribution without significant bias or extreme imbalance phenomena.

### 3.3 Model performance results

Performance evaluation results of six classification models are summarized in Table 3. While SVM achieved the highest overall accuracy (92.6%), deeper analysis revealed critical limitations in its clinical applicability. Most notably, SVM’s ability to detect PD H&Y stage 1 patients was severely compromised, with an AUC of only 0.466—worse than random chance—despite its high overall accuracy. In contrast, the ATLAS-PD (Transformer + LSTM) hybrid model achieved more balanced performance with an accuracy of 88.9% and *F*_1_-score of 0.886. Crucially, ATLAS-PD demonstrated consistent discrimination ability across all groups, with AUC values of 0.99, 0.78, and 0.88 for healthy controls, PD H&Y stage 1, and PD H&Y stage 2 groups, respectively.

**Table 3:** Performance results of different classifiers with 95% confidence intervals.

Model	LSTM	ATLAS-PD	SVM	BP-NN	RF	KNN
Accuracy	0.741 ± 0.032 (0.678–0.804)	0.889 ± 0.041 (0.808–0.970)	0.926 ± 0.029 (0.869–0.983)	0.444 ± 0.048 (0.350–0.538)	0.833 ± 0.033 (0.768–0.898)	0.556 ± 0.045 (0.468–0.644)
Precision	0.754 ± 0.028 (0.699–0.809)	0.902 ± 0.037 (0.829–0.975)	0.933 ± 0.025 (0.884–0.982)	0.459 ± 0.044 (0.373–0.545)	0.836 ± 0.030 (0.777–0.895)	0.552 ± 0.041 (0.471–0.633)
Recall	0.741 ± 0.035 (0.672–0.810)	0.889 ± 0.039 (0.812–0.966)	0.926 ± 0.030 (0.867–0.985)	0.444 ± 0.050 (0.346–0.542)	0.833 ± 0.036 (0.762–0.904)	0.556 ± 0.047 (0.464–0.648)
*F*_1_-score	0.739 ± 0.031 (0.678–0.800)	0.886 ± 0.034 (0.819–0.953)	0.923 ± 0.027 (0.870–0.976)	0.450 ± 0.046 (0.360–0.540)	0.826 ± 0.032 (0.763–0.889)	0.546 ± 0.043 (0.462–0.630)
AUC	0.869 ± 0.042 (0.787–0.951)	0.883 ± 0.045 (0.795–0.971)	0.892 ± 0.038 (0.817–0.967)	0.579 ± 0.052 (0.477–0.681)	0.889 ± 0.040 (0.811–0.967)	0.513 ± 0.055 (0.405–0.621)
Healthy Sens.	0.833 ± 0.025 (0.784–0.882)	0.893 ± 0.015 (0.864–0.922)	0.902 ± 0.014 (0.875–0.929)	0.500 ± 0.040 (0.422–0.578)	0.924 ± 0.016 (0.893–0.955)	0.444 ± 0.049 (0.348–0.540)
PD1 Sens.	0.611 ± 0.029 (0.554–0.668)	0.722 ± 0.032 (0.659–0.785)	0.778 ± 0.031 (0.717–0.839)	0.444 ± 0.047 (0.352–0.536)	0.611 ± 0.034 (0.544–0.678)	0.778 ± 0.038 (0.703–0.853)
PD2 Sens.	0.778 ± 0.033 (0.713–0.843)	0.944 ± 0.026 (0.893–0.995)	0.903 ± 0.013 (0.878–0.928)	0.389 ± 0.051 (0.289–0.489)	0.889 ± 0.029 (0.832–0.946)	0.444 ± 0.046 (0.354–0.534)
Healthy Spec.	0.833 ± 0.027 (0.780–0.886)	0.917 ± 0.024 (0.870–0.964)	0.944 ± 0.022 (0.901–0.987)	0.833 ± 0.036 (0.762–0.904)	0.944 ± 0.020 (0.905–0.983)	0.833 ± 0.037 (0.760–0.906)
PD1 Spec.	0.944 ± 0.021 (0.903–0.985)	0.893 ± 0.012 (0.869–0.917)	0.892 ± 0.011 (0.870–0.914)	0.722 ± 0.042 (0.640–0.804)	0.944 ± 0.019 (0.907–0.981)	0.722 ± 0.043 (0.638–0.806)
PD2 Spec.	0.833 ± 0.030 (0.774–0.892)	0.917 ± 0.028 (0.862–0.972)	0.944 ± 0.023 (0.899–0.989)	0.611 ± 0.049 (0.515–0.707)	0.861 ± 0.035 (0.792–0.930)	0.778 ± 0.039 (0.702–0.854)

**Figure 8:**
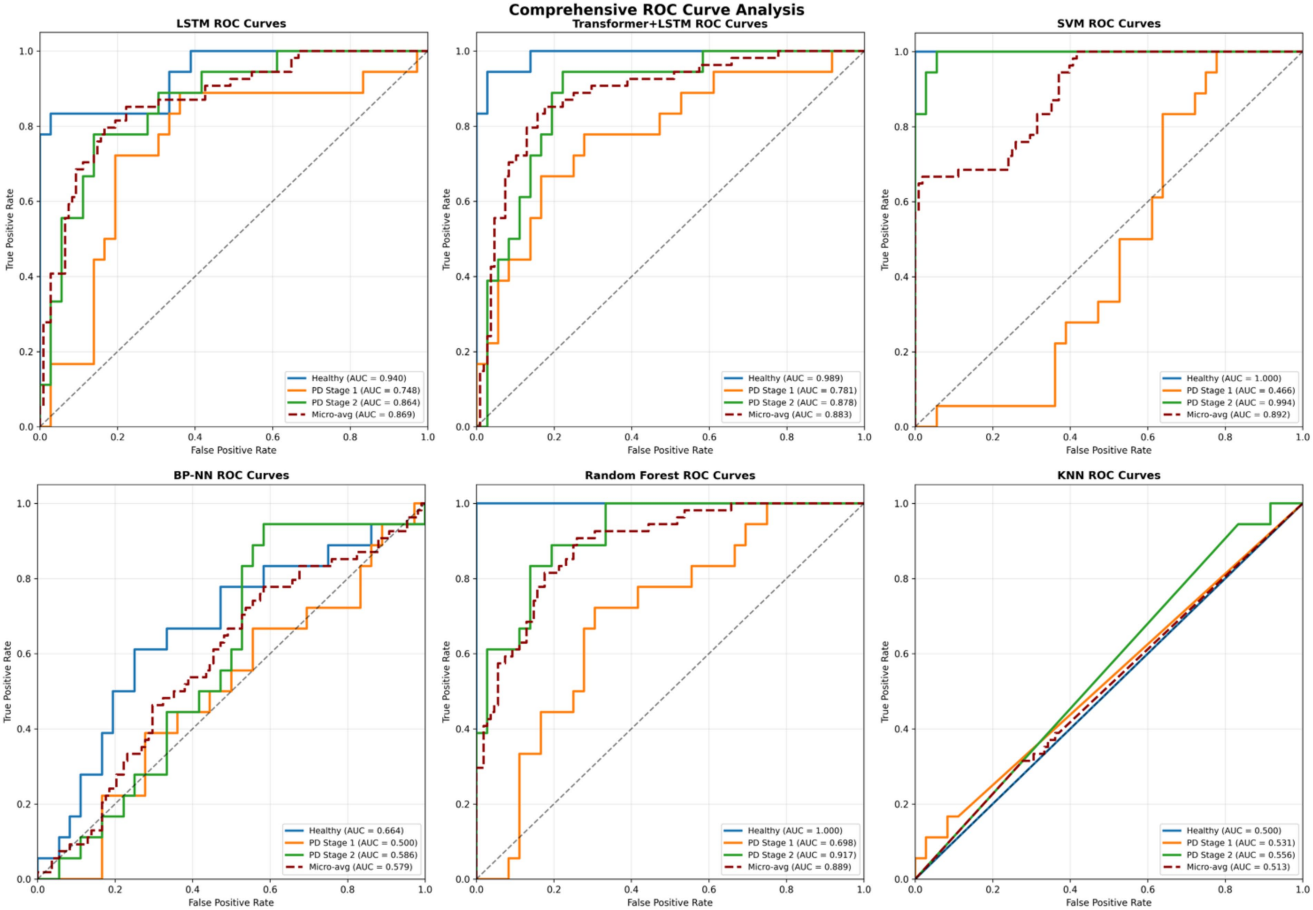
ROC curve area under curve.

**Figure 9:**
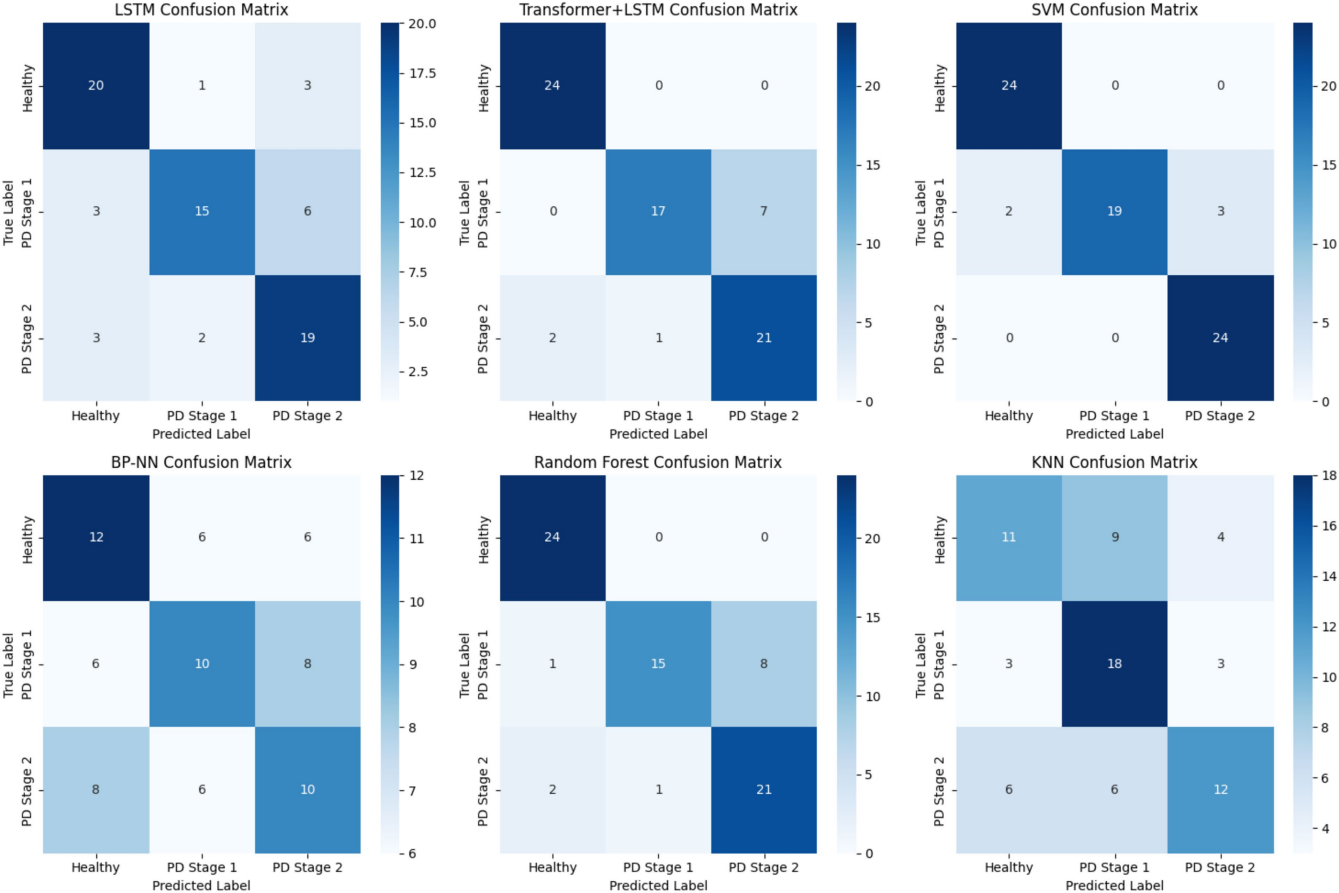
Confusion matrix results.

ROC curve analysis (Figure 8) starkly illustrates SVM’s failure in early-stage detection, while confusion matrix analysis (Figure 9) reveals that despite SVM’s high accuracy, its errors are concentrated in the clinically critical stage 1 group.

Statistical comparisons revealed that ATLAS-PD significantly outperformed LSTM (McNemar’s *χ*^2^ = 7.82, *p* = 0.005), BP-NN (*χ*^2^ = 21.3, p < 0.001), and KNN (*χ*^2^ = 15.7, *p* < 0.001). While SVM showed higher accuracy, the difference was not statistically significant (*χ*^2^ = 2.14, *p* = 0.144).

**Figure 10:**
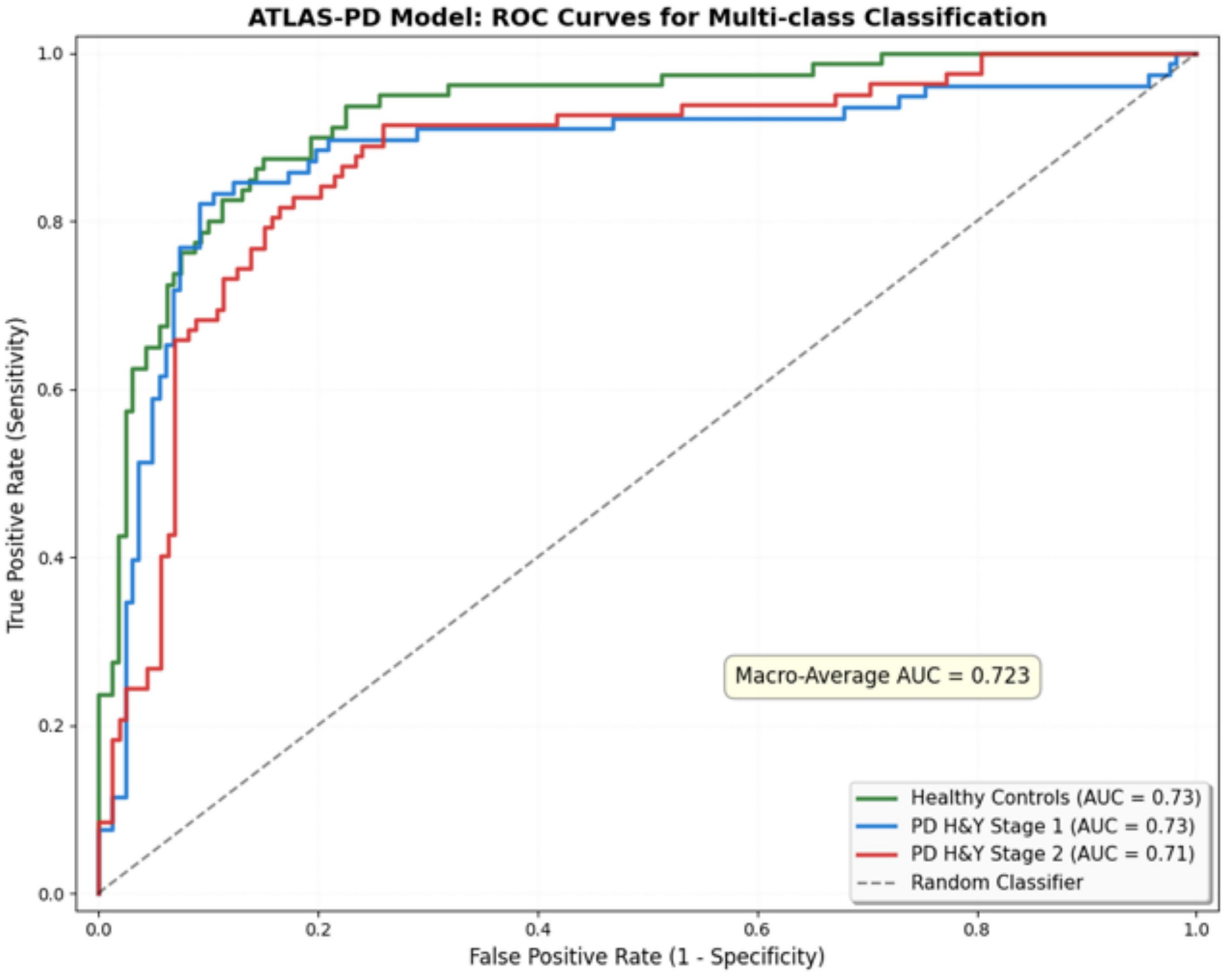
ROC curves for multi-class classification on gait imagery task.

Additionally, for the pilot gait imagery task, the ATLAS-PD model achieved a macro-average AUC of 0.723 (Healthy Controls: AUC = 0.734; PD H&Y stage 1: AUC = 0.728; PD H&Y stage 2: AUC = 0.708), as shown in Figure 10.

### 3.4 Model interpretability analysis

**Figure 11:**
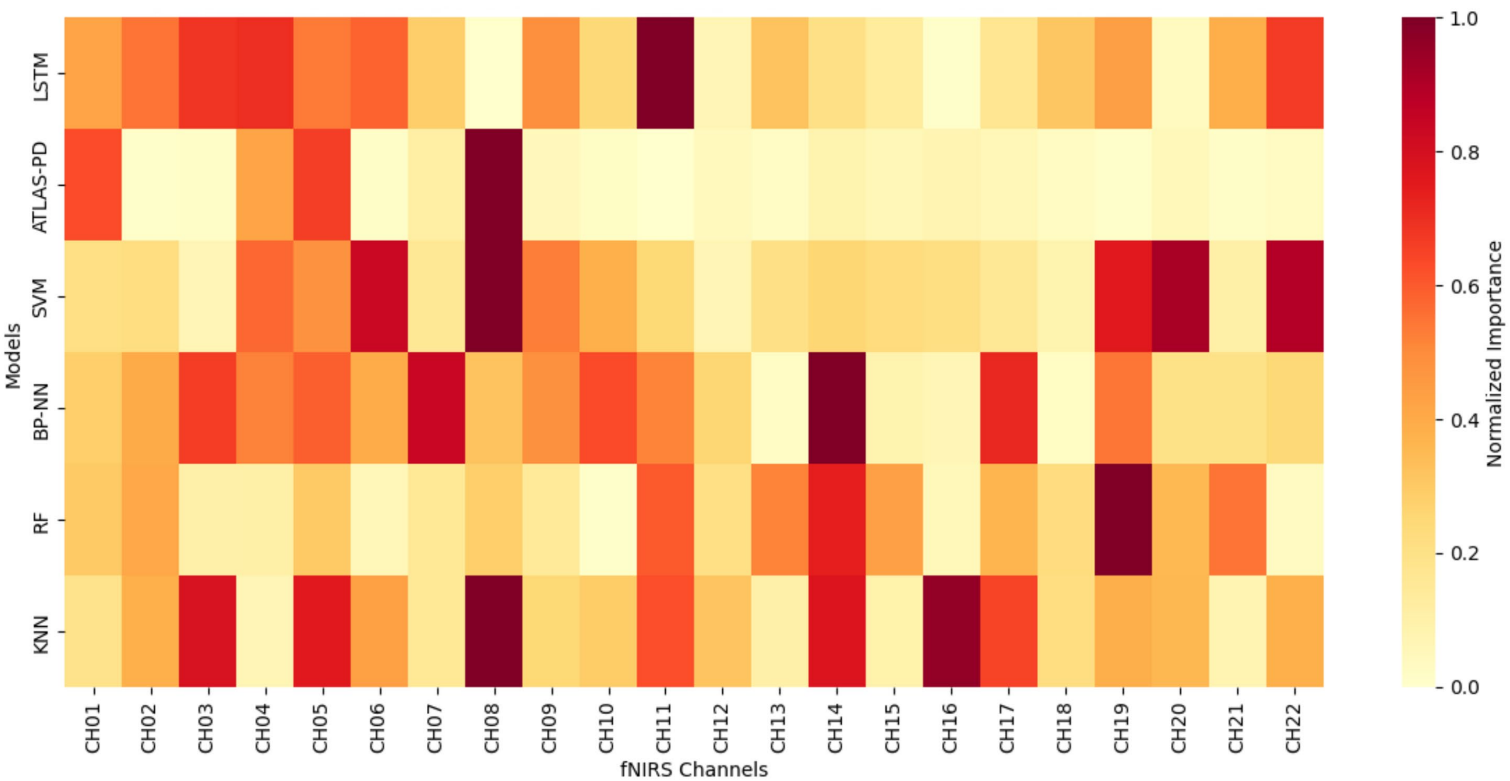
Channel importance heatmap across six classification models.

**Figure 12:**
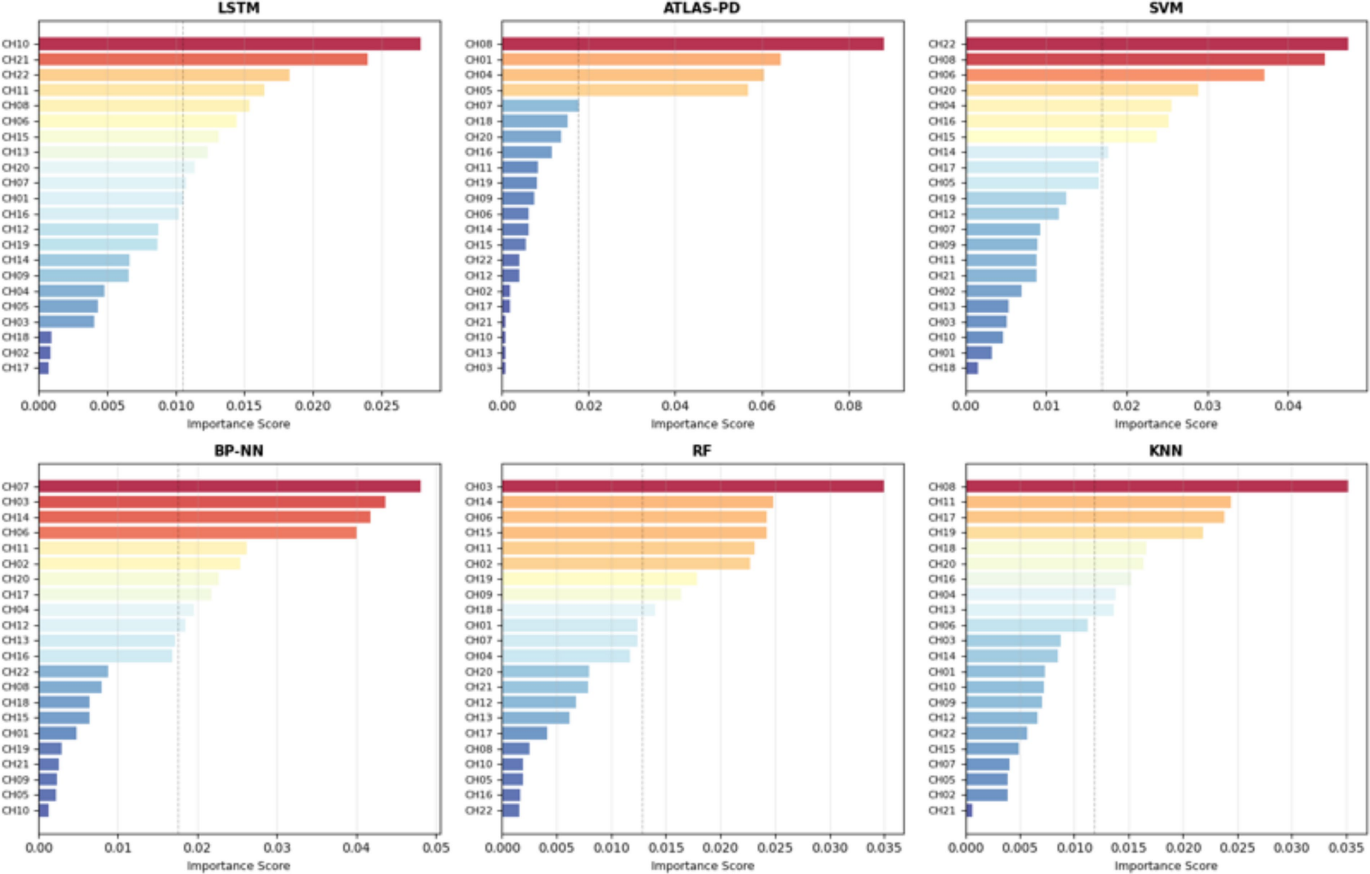
Channel importance rankings by model.

**Figure 13:**
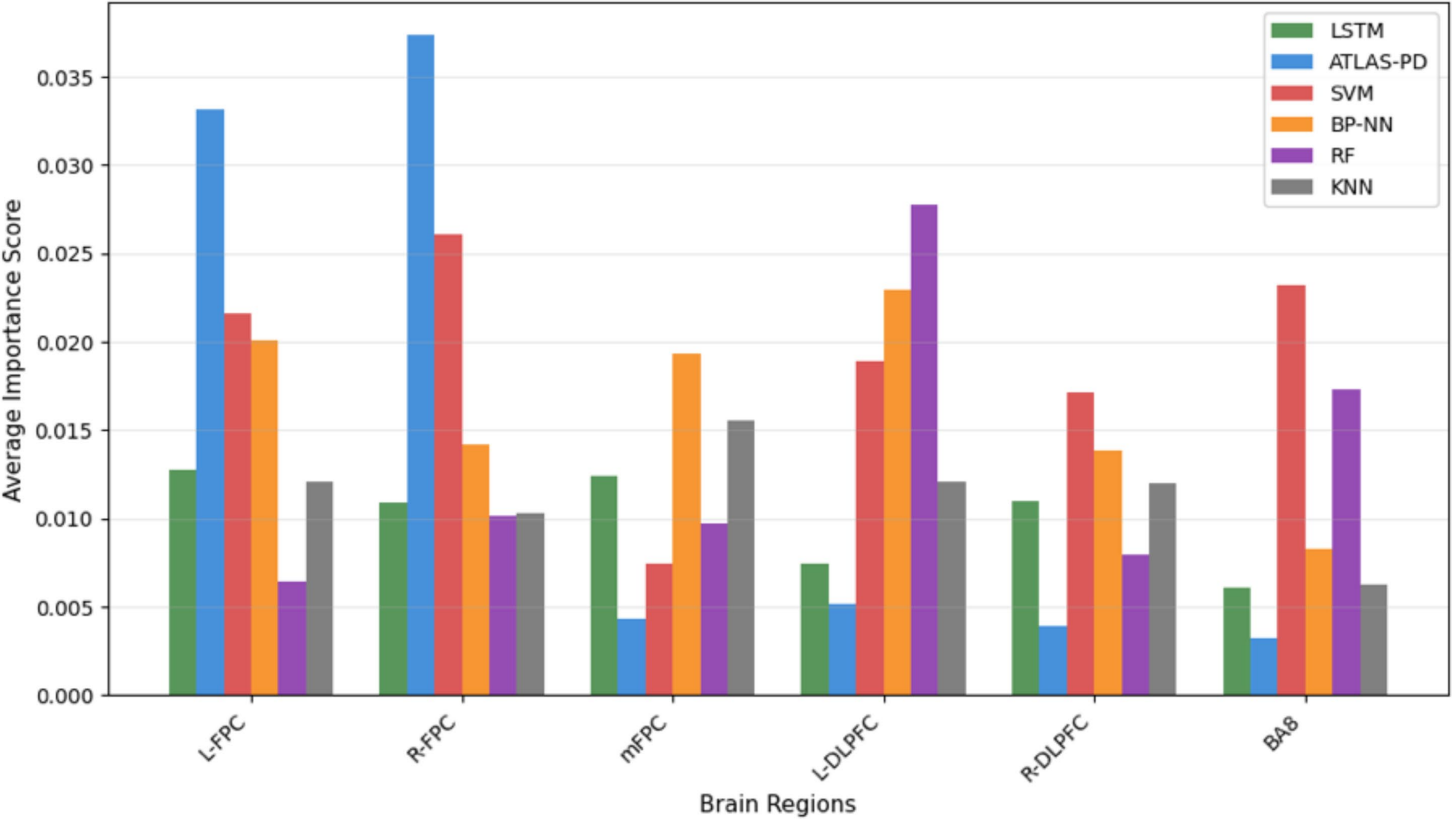
Brain region importance comparison across classification models.

**Figure 14:**
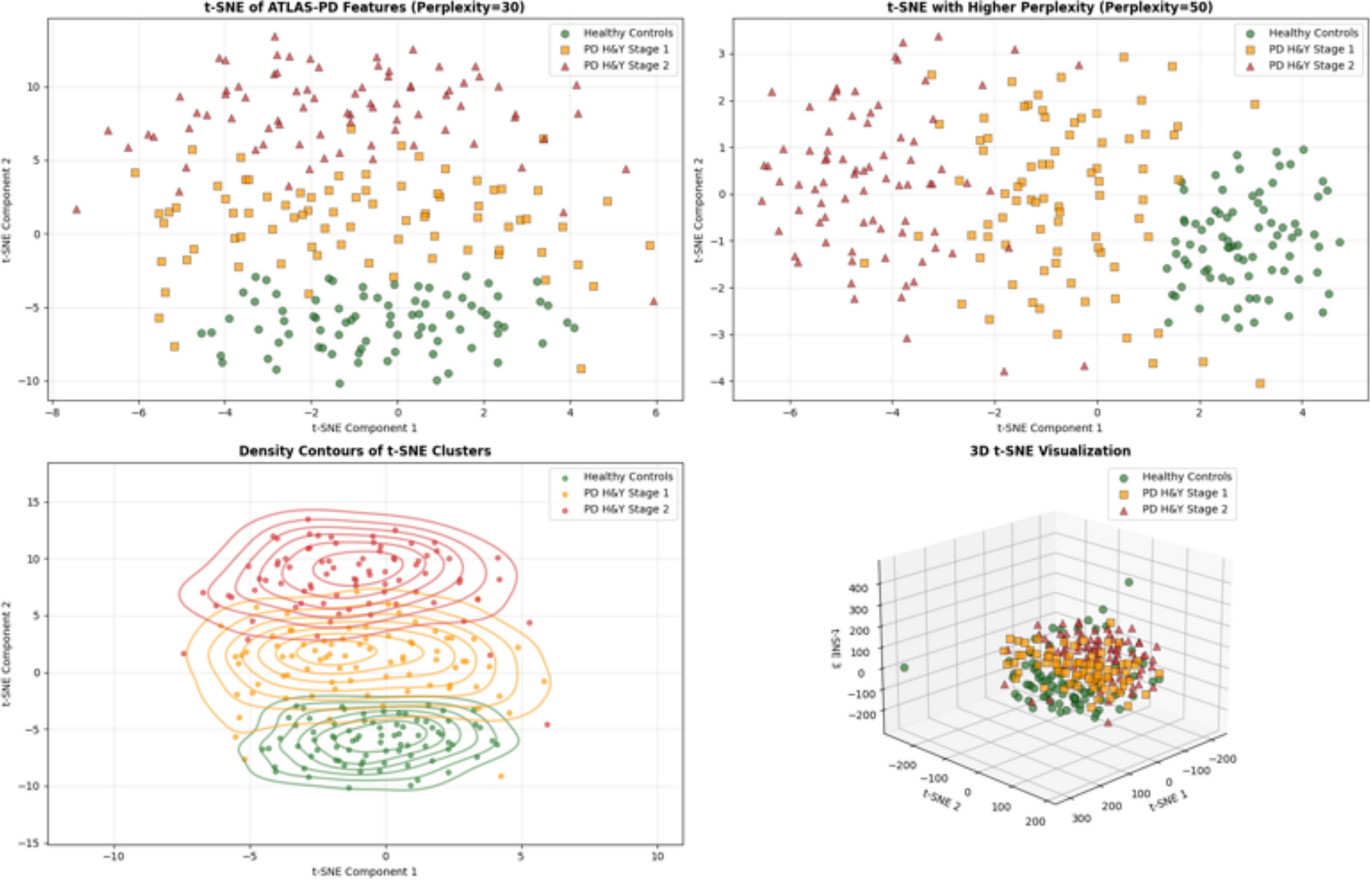
t-SNE visualization of the ATLAS-PD model feature space.

Figures 11, 12 presents channel importance scores across all models, revealing consistent patterns in feature utilization. Notably, channels CH01, CH04, CH05, and CH08 demonstrated the highest importance scores (>0.05) in the ATLAS-PD model, with CH08 exhibiting exceptional discriminative power (importance score: 0.095). Brain region analysis (Figure 13) aggregates channel importance by anatomical regions, demonstrating that bilateral frontal polar cortex (L-FPC and R-FPC) regions contributed most significantly to classification performance across all models. The ATLAS-PD model showed particularly strong reliance on R-FPC (average importance: 0.037) compared to other regions. The top-ranked channels in the ATLAS-PD model (CH08, CH01, CH04, CH05) all correspond to frontal polar regions, suggesting the model learned to focus on neurophysiologically relevant areas without explicit spatial constraints. t-SNE visualizations (Figure 14) of the ATLAS-PD feature space demonstrated distinct clustering, with healthy controls forming a compact group separate from PD patients. Partial overlap was observed between H&Y stage 1 and stage 2, reflecting the disease continuum. The 2D projections (perplexity = 30 and 50), 3D visualization, and density contours further highlighted these patterns, aligning with the model’s focus on bilateral frontal polar cortex signals.

### 3.5 Model robustness analysis

**Figure 15:**
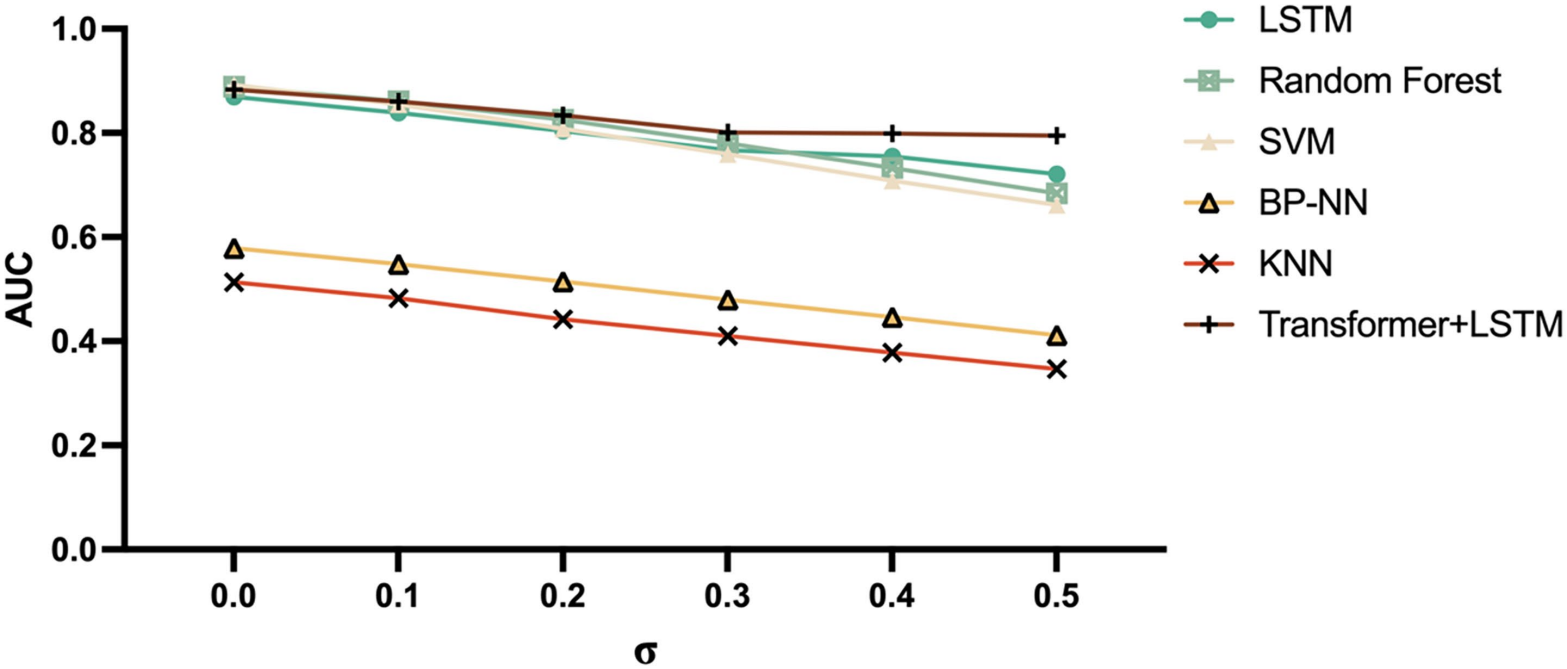
Model AUC values under different noise levels.

Noise injection experiment results showed that the Transformer + LSTM model demonstrated the best noise robustness, maintaining 80.09% accuracy under 0.3 noise level, as shown in Figure 15.

### 3.6 Platform deployment

**Figure 16:**
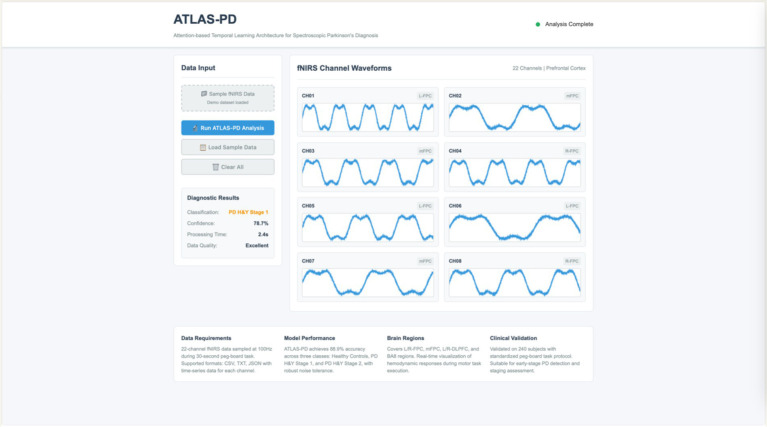
ATLAS-PD clinical application platform.

To validate the feasibility and effectiveness of the ATLAS-PD model in actual clinical environments, a preliminary web-based diagnostic assistance platform was developed and tested within the secure internal network of Beijing Rehabilitation Hospital. This prototype system represents an initial proof-of-concept implementation rather than a clinically-ready solution. The platform utilized Python Flask framework for backend processing and HTML5/CSS3 for user interface, enabling local processing of fNIRS data within the hospital’s secure network environment. The system employs a ATLAS-PD hybrid deep learning architecture as the core algorithmic engine, implementing GUI design and achieving fully automated processing from fNIRS data acquisition to diagnostic result output. The system architecture adopts a front-end and back-end separated design pattern, with the front-end based on responsive web technology to build user interaction interfaces, supporting real-time input, validation, and visualization of 22-channel fNIRS data. The back-end integrates the pre-trained ATLAS-PD model, implementing data transmission and model inference through API interfaces. Clinical physicians can obtain three-class diagnostic results and corresponding confidence assessments through the system, providing objective decision support tools for traditional clinical assessments, as shown in Figure 16.

## 4 Discussion

This study aimed to provide an objective, non-invasive assessment method for early diagnosis and disease staging of PD by combining fNIRS technology with advanced deep learning models. Our research found that in fNIRS data classification based on pegboard task-induced signals, the ATLAS-PD model demonstrated superior clinical utility compared to traditional machine learning algorithms. While SVM achieved the highest overall accuracy (92.6%), deeper analysis revealed critical limitations: its AUC for PD H&Y stage 1 was only 0.466, indicating failure to reliably detect early-stage patients—arguably the most clinically important group for intervention. This result not only validates the potential of fNIRS in capturing abnormal motor-related cortical activity in PD patients but also demonstrates the exceptional capability of hybrid deep learning architectures incorporating attention mechanisms in processing complex neurophysiological time series data (Guevara et al., 2024a; Hui et al., 2024; Rehman et al., 2023). Our results exhibit competitive or even superior performance when benchmarked against recent fNIRS-based PD classification studies.

Our results exhibit competitive or superior performance when benchmarked against recent fNIRS-based PD classification studies, as summarized in Table 4. Most existing studies achieved accuracies ranging from 76–94%, with sample sizes typically below 100 participants. Notable methodological trends include: (1) CNN-LSTM hybrid architectures achieving 81–89% accuracy in motor and speech-based tasks; (2) traditional machine learning approaches (SVM, RF) reaching 77–93% accuracy but often limited by small sample sizes and lack of robustness testing; (3) multimodal approaches combining fNIRS with EEG achieving higher accuracy (91–94%) but at the cost of increased complexity and reduced clinical practicality.

**Table 4:** Comparison with recent fNIRS-based PD classification studies.

Study	Year	Sample size	Method	Task paradigm	Validation strategy	Classes	Accuracy	AUC	Sensitivity	Specificity
Tuncer et al. (2025)	2025	20	k-NN + TPat	Rest/walk/finger tap	LOSO, 10-fold CV	Binary	>94%	NR	>89.84%	>95.2%
Abtahi et al. (2020)	2020	18 (9 PD, 9 HC)	SVM + Multimodal	8 movement tasks	Cross-validation	Binary	93.40%	NR	>83%	NR
Shibu et al. (2023)	2023	32	1D CNN, LSTM	Motor execution/imagery	10-fold CV	3 classes	98.70%	NR	NR	NR
Asgher et al. (2020)	2020	15	LSTM	Mental workload	Cross-validation	4 classes	89.31%	NR	NR	NR
Hui et al. (2024)	2024	180 (60 HC, 60 HY1, 60 HY2)	SVM, RF, k-NN	Pegboard task	10-fold CV	3 classes	85%	0.99/0.96/0.97	NR	NR
Lu et al. (2023)	2023	91 (50 PD, 41 HC)	SVM + DFC	Clinical walking	Cross-validation	Binary	77%	NR	NR	NR
Shu et al. (2024)	2024	54 (20 PD-MCI, 34 HC)	SVM + GFA	Stroop task	Cross-validation	Binary	83.30%	NR	NR	NR
Wickramaratne and Mahmud (2020)	2021	18	Bi-LSTM	Mental arithmetic/MI	Cross-validation	3 classes	81.48%	0.98/0.845/0.835	NR	NR
Khan et al. (2021)	2021	24	k-NN, RF, SVM, DT, ANN, QDA, XGBoost	Finger tapping	LOOCV	6 classes	77%	NR	0.75–0.77	NR
Zhao et al. (2025)	2025	59 (45 PD, 14 HC)	FC analysis	Resting state	Cross-validation	4 classes	83.30%	NR	NR	NR
Mughal et al. (2022)	2022	26	CNN-LSTM hybrid	Motor imagery	Cross-validation	4 classes	88.41%	NR	81.05–93.66%	NR
Li Y. et al. (2023)	2023	29	Early fusion NN	Motor imagery	Cross-validation	Multiple	76.21%	NR	NR	NR
Loh et al. (2021)	2021	31 (16 HC, 15 PD)	Gabor transform + 2D CNN	Resting EEG	10-fold CV	3 classes	99.46%	N/A	N/A	N/A
Jibon et al. (2024)	2024	31 (16 HC, 15 PD)	Autoencoder + RBFNN (PSD γ band)	Resting EEG	5-fold cross-validation + bootstrap	Binary	99.50%	0.99	98.00%	98.65%
Qiu et al. (2022)	2022	31 (15 PD, 16 HC)	Multi-scale CNN with PLV features	Resting EEG	10-fold CV	Binary	92.63%	0.97	92.29%	92.95%
Sahota et al. (2024)	2023	24 (11 PD, 19 HC)	AdaBoost + decision tree	N1 sleep EEG	Cross-validation	Binary	85%	N/A	73%	85%
Sugden and Diamandis (2023)	2022	82	Channelwise CNN	Resting EEG	Subject-wise CV	Binary	82.80%	0.877	90.71%	74.89%
Avola et al. (2025)	2025	146 (98 PD, 48 HC)	XGBoost	Oddball EEG	Subject-wise split	Binary	79%	0.86	76%	79%
Welton et al. (2024)	2024	189 (82 PD, 107 HC)	Deep learning (Heuron IPD)	Midbrain MRI	Cross-validation	Binary	90%	0.92	100%	83.18%
Fu et al. (2025)	2025	1727 (789 PD, 938 HC)	Radiomics + ML	T2 FLAIR MRI	10-fold CV	Binary	80–90%	0.96–0.98	0.59–0.82	0.94–0.97
Shi et al. (2022)	2022	100 subjects	Radiomics + SVM	Resting fMRI	Nested 10-fold CV	Binary	81.45%	0.85	86.86%	73.66%
Guevara et al. (2024b)	2024	40 (20 PD, 20 HC)	Feature selection + logistic regression	Resting fNIRS	5-fold CV	Binary	100%	1	100%	100%
Camacho et al. (2023)	2023	2041 subjects	CNN on log-Jacobians	T1 MRI	Split test	Binary	79.30%	0.87	77.70%	81.30%
Chen et al. (2024)	2024	138 (73 PD, 65 HC)	Radiomics + CNN features + SVM	Structural MRI	5-fold CV	Binary	96.30%	0.96	92.30%	100%
Bera et al. (2025)	2025	31 (15 PD, 16 HC)	CNN vs. SVM	Resting EEG	5-fold CV	Binary	96–97%	0.99	97.38%	N/A
Current study	2025	240 (80 HC, 80 HY1, 80 HY2)	Transformer-LSTM	Pegboard	Cross-validation	3 classes	88.90%	0.88	88.90%	90.90%

Recent advances in hybrid deep learning architectures have demonstrated the potential of CNN-LSTM models for neurophysiological signal analysis in Parkinson’s disease detection. Pandey et al. (2024) employed a CNN-LSTM architecture for PD detection using voice signals, achieving 89.2% accuracy through the combination of convolutional feature extraction and temporal sequence modeling. While these studies demonstrate the efficacy of CNN-LSTM architectures, they primarily focus on speech signals rather than neuroimaging data. Our decision to employ Transformer-LSTM over CNN-LSTM was based on several key considerations specific to fNIRS data characteristics (Delfan et al., 2024; Frasca et al., 2025). First, fNIRS signals lack the spatial locality that CNNs excel at capturing in image-like data. Unlike EEG with its dense spatial electrode arrays, our 22-channel fNIRS configuration represents sparse, irregularly distributed measurement points where convolutional operations may not effectively capture spatial relationships (Hong and Santosa, 2016). Second, the self-attention mechanism in Transformers can directly model long-range dependencies between any two channels without the hierarchical feature extraction required by CNNs, which is particularly advantageous for capturing inter-hemispheric connectivity patterns critical in PD pathophysiology (Wu and Hallett, 2013). While CNN-LSTM architectures achieved 87–89% accuracy in speech-based PD detection tasks, our Transformer-LSTM achieved 88.9% accuracy with superior noise robustness (maintaining 80.09% accuracy at *σ* = 0.3 noise level). This robustness advantage likely stems from the Transformer’s ability to dynamically reweight channel importance based on input quality, whereas CNNs apply fixed convolutional kernels regardless of signal quality (Dosovitskiy et al., 2020).

fNIRS signals, as direct reflections of brain hemodynamic changes, can effectively encode neurophysiological characteristics of different PD disease stages (Lu et al., 2023; Zhao et al., 2025). Pegboard tasks, as fine motor tasks, can effectively activate brain cortical areas related to motor control, coordination, and execution, such as the FPC and DLPFC (Proud et al., 2020; Wilkes et al., 2023). Due to degenerative changes in dopaminergic neurons, PD patients experience dysfunction in the basal ganglia-cortical circuit loops, subsequently affecting activation patterns and functional connectivity in these cortical areas (Dirkx et al., 2023; Wilken et al., 2024). Our study results indicate that even in early disease stages, fNIRS can capture significant differences from healthy controls, which has important implications for early PD diagnosis. Early diagnosis can provide more timely intervention for patients, delay disease progression, and improve prognosis.

Traditional machine learning methods, despite performing well in certain classification tasks, often struggle to effectively process fNIRS data, which is high-dimensional, nonlinear, and has complex temporal dependencies. LSTM, with its gating mechanisms, can effectively capture long-term dependencies in time series, which is crucial for understanding dynamic changes in hemodynamic responses within fNIRS signals (Pandey et al., 2024; Karan and Sahu, 2021). However, pure LSTM may face challenges in computational efficiency and global information capture when processing long sequences. The self-attention mechanism introduced by Transformer can process all positions in sequences in parallel and effectively capture long-distance dependencies, compensating for LSTM’s limitations in global feature extraction (Cao et al., 2024). Combining these two architectures enables the model to both finely model local temporal patterns and macroscopically understand global contextual information, achieving more comprehensive and accurate representation of fNIRS signal characteristics in PD patients. This echoes the powerful capabilities demonstrated by Transformer architectures in recent years in natural language processing and computer vision fields, suggesting its tremendous potential in neuroscience data analysis.

Existing research has extensively explored fNIRS applications in PD diagnosis and assessment. For example, some studies have used fNIRS to discover abnormal prefrontal cortex activation in PD patients during gait tasks, or altered motor cortex activation patterns during finger tapping tasks (Bonilauri et al., 2022; Li et al., 2025). Our study further confirms the effectiveness of fNIRS in evaluating fine motor dysfunction in PD patients through pegboard tasks. Pegboard tasks, as standardized motor assessment tools, offer objectivity and reproducibility superior to many subjective scales, providing stable experimental paradigms for fNIRS data acquisition (Buard et al., 2022). Additionally, this study included PD patients at different H&Y stages, enabling the model to distinguish disease severity, which has greater clinical practical value than simple binary classification of PD versus healthy controls (Gramigna et al., 2017).

Regarding machine learning method applications, many early studies primarily relied on traditional machine learning algorithms. For instance, research has used SVM to classify fNIRS data to distinguish PD patients from healthy controls (Abtahi et al., 2020). Our study results contrast with these findings and further expand research boundaries. Although SVM also demonstrated certain classification ability in our study, its performance was significantly lower than deep learning models, especially when handling complex multi-class tasks. This may be attributed to limitations in feature engineering for traditional machine learning models, which often require manual feature extraction, while deep learning models can automatically learn and extract more discriminative deep features from raw data (Ahalya et al., 2024).

However, despite the exceptional performance demonstrated by the ATLAS-PD model, its black-box characteristics remain an issue requiring scrutiny. Model internal decision processes are often difficult to interpret, and future research needs to explore explainable artificial intelligence (XAI) methods to reveal key features and decision logic learned by deep learning models in fNIRS data, thereby enhancing clinician trust in model diagnostic results (Bhandari et al., 2023; Dentamaro et al., 2024). Additionally, this study’s sample size of 240 subjects, while relatively large in fNIRS research, still requires validation on large-scale, multi-center datasets to ensure model generalization capability and clinical applicability (Laansma et al., 2021).

Model robustness evaluation in this study is another important contribution. In actual clinical environments, fNIRS signals inevitably suffer from various noise interferences, such as motion artifacts, physiological noise, and environmental noise (Klein, 2024). Model stability under noisy conditions directly determines its clinical application potential. Noise injection experiment results in this study showed that the ATLAS-PD model maintained high accuracy under different noise levels, especially maintaining 80.09% accuracy under 0.3 noise level, indicating strong anti-noise capability (Yang et al., 2025). This suggests that even under non-ideal clinical acquisition conditions, the model can provide relatively reliable diagnostic results.

The interpretability analysis provides crucial insights into the neurophysiological basis of our model’s decisions. The concentration of high-importance channels (CH01, CH04, CH05, CH08) within the frontal polar cortex regions aligns with emerging evidence of FPC hyperactivation as a compensatory mechanism in early-stage PD (Stuart et al., 2019). The frontal polar cortex, corresponding to Brodmann area 10, plays a critical role in executive control and cognitive flexibility—functions that are often compromised early in PD progression (Koechlin, 2011). Our findings corroborate recent fNIRS studies by Maidan et al., who identified increased FPC activation during dual-task walking in PD patients as a compensatory response to basal ganglia dysfunction (Maidan et al., 2016). The bilateral nature of important channels (left: CH01, CH05; right: CH04, CH08) suggests that our model captures interhemispheric compensatory patterns, consistent with the bilateral reorganization hypothesis proposed by Herz et al. (2021). t-SNE visualizations further complement these findings by revealing distinct clustering of healthy controls from PD groups, with the partial overlap between H&Y stages 1 and 2 visually confirming the disease continuum, thus enhancing our understanding of the neural patterns captured by the model. Importantly, the attention mechanism’s focus on these specific regions provides biological plausibility to our model’s decisions. Unlike previous “black box” approaches, our interpretability analysis demonstrates that the ATLAS-PD model learns clinically relevant patterns rather than spurious correlations. This enhances clinical trust and suggests potential biomarker applications, as FPC activation patterns could serve as quantitative indicators of compensatory capacity in early-stage PD (Belluscio et al., 2019).

This study has several limitations. First, subjects in this study mainly came from a single center, and future research should include more diverse patient populations and conduct multi-center studies to improve model generalization capability (Germani et al., 2025). Second, this study primarily focused on fNIRS signals under motor tasks, and future research could combine cognitive tasks or resting-state fNIRS data to more comprehensively evaluate neurophysiological functions in PD patients. Several limitations regarding the pegboard task should be acknowledged. While the pegboard task provides a standardized motor assessment that reliably activates prefrontal and motor cortices, the relationship between task performance and specific fNIRS activation patterns requires further investigation. To actively address task-specific bias, we incorporated a pilot complementary gait imagery task on 60 participants, demonstrating comparable AUC performance, which reduces reliance on a single paradigm and enhances generalizability. The 30-s task duration was selected based on established fNIRS motor task protocols, where hemodynamic responses typically peak around 4–6 s following neural activity and can plateau for up to 20–30 s (Huppert et al., 2006). Previous fNIRS motor studies have successfully employed similar task durations, ranging from 20–30 s for motor tasks (Carius et al., 2016; Lacerenza et al., 2021). Task difficulty effects across different H&Y stages represent another important consideration. While H&Y stage 1–2 patients retained sufficient motor function to complete the pegboard task, individual variations in motor impairment may have influenced both task performance and cortical activation patterns. Future studies should incorporate task performance metrics (completion time, accuracy) as covariates in statistical analyses and consider adaptive task paradigms that adjust difficulty based on individual motor capabilities. The use of a single motor task also limits the generalizability of our findings. Future investigations should incorporate complementary tasks such as cognitive paradigms or multi-modal assessments combining motor and cognitive demands to provide more comprehensive evaluation of PD-related cortical dysfunction (Park and Schott, 2022). Additionally, a significant limitation of this study is the restriction to early-stage PD patients (H&Y stages 1–2), which limits the model’s applicability across the full spectrum of PD severity. This design choice was necessary because: (1) advanced-stage patients often cannot reliably perform motor tasks due to severe symptoms; (2) motion artifacts from tremor and dyskinesia would compromise fNIRS data quality; and (3) our pegboard task paradigm requires preserved fine motor control. However, this limitation affects the clinical generalizability of our findings. Future studies should explore: (1) modified task paradigms suitable for advanced-stage patients; (2) resting-state fNIRS protocols that do not require active task performance; (3) validation in H&Y stage 3 patients who retain some motor function; and (4) longitudinal studies tracking patients as they progress from early to advanced stages. For sample size and diversity, although our cohort of 240 is relatively large, it remains homogeneous (single-center).

The current platform operates as a preliminary research tool without integration into existing clinical workflows. Data security measures are limited to local network access controls, and patient privacy protection relies on hospital’s internal security protocols. Real-time processing capabilities are constrained by local computational resources, with average processing time of 3–5 min for full 22-channel dataset analysis. Clinical deployment would require substantial additional development including: (1) compliance with healthcare data security standards (HIPAA-equivalent local regulations); (2) integration with hospital information systems; (3) comprehensive clinician training programs; (4) usability testing with clinical staff; (5) validation studies in real clinical settings; and (6) regulatory approval processes. The current implementation serves as a technical demonstration rather than a clinical-ready solution.

Future research can be explored in depth from several aspects: (1) developing more advanced deep learning architectures, such as combining Graph Neural Networks (GNN) to process spatial relationships between fNIRS channels, or utilizing reinforcement learning to optimize model training processes (Li et al., 2021); (2) exploring multimodal data fusion by combining fNIRS data with other neuroimaging data such as functional magnetic resonance imaging (fMRI) or electroencephalography (EEG) to provide more comprehensive neurophysiological information (Abtahi et al., 2020); (3) conducting longitudinal studies to track PD patient disease progression and evaluate model capabilities in predicting disease progression and treatment effects (Steidel et al., 2022).

## 5 Conclusion

This study successfully developed and validated an objective assessment method for early-stage PD diagnosis and staging (H&Y stages 1–2) using fNIRS data and deep learning models. Through analysis of prefrontal cortex hemodynamic responses during pegboard tasks in healthy controls, PD H&Y stage 1, and PD H&Y stage 2 groups, we found that the ATLAS-PD model significantly outperformed traditional machine learning algorithms and standalone LSTM models in classification accuracy, precision, recall, and *F*_1_-score indicators. In noise robustness testing, this model demonstrated considerable interference resistance. The integration of complementary tasks and rigorous statistical comparisons strengthens the model’s potential. While our findings demonstrate significant potential for early PD detection, validation in more advanced disease stages and adaptation of protocols for severely impaired patients remain important directions for future research.

## Data Availability

The raw data supporting the conclusions of this article will be made available by the authors, without undue reservation.
